# Scurvy in a Child Presenting With a Limp and Elevated Inflammatory Markers: A Case Report

**DOI:** 10.7759/cureus.62101

**Published:** 2024-06-10

**Authors:** Dan Rittenhouse, Alexander Daly

**Affiliations:** 1 Pediatrics, Penn State College of Medicine, Hershey, USA; 2 Pediatrics, Penn State Health Children's Hospital, Hershey, USA

**Keywords:** vitamin deficiency, pediatric, vitamin c, child, scurvy, inflammatory markers

## Abstract

Scurvy is a rare diagnosis in resource-rich countries, but cases have been documented in the United States in special populations of pediatric patients at increased risk of micronutrient deficiency such as those with autism spectrum disorder, developmental delay, or eating disorders. We discuss a seven-year-old female with autism spectrum disorder who presented with a limp and refusal to ambulate and elevated inflammatory markers on initial laboratory evaluation. Given her highly selective diet and malnutrition, we made a provisional diagnosis of scurvy and started treatment-dose vitamin C, which led to a significant improvement in her ambulatory function. Plasma vitamin C was ultimately undetectable. She was discharged with vitamin C supplementation and referred to a feeding clinic to address her malnutrition and selective eating.

## Introduction

Scurvy is a clinical manifestation of vitamin C deficiency. Vitamin C acts as an essential cofactor for enzymes involved in the synthesis of collagen but also appears to increase the transcription of collagen types I and III in fibroblasts. Deficiency of vitamin C can cause the development of fragile skin and blood vessels due to poor collagen synthesis [1]. In the male pediatric population in the United States, the 10th to 90th percentile of serum vitamin C levels range from 43.6 μmol/L to 108.0 μmol/L for those aged 6-11 years and 20.6 μmol/L to 85.1 μmol/L for ages 12-19 [2]. For pediatric females, the 10th to 90th percentile range for serum vitamin C is 36.4-106.9 μmol/L for patients aged 6-11 years and 19.0-91.6 μmol/L for ages 12-19 [2].

In general, clinical features of vitamin C deficiency appear when levels drop below 11 μmol/L [3]. Scurvy may initially present with constitutional symptoms such as fevers, fatigue, malaise, lethargy, or anorexia. However, patients may also complain of bleeding gums, rashes, easy bruising, bone and joint aches, or limp. On physical examination, patients with scurvy may have periodontal disease, gingivitis, petechiae, perifollicular hemorrhages, alopecia, corkscrew hairs, or joint swelling [1,4]. Inflammatory markers can be elevated in scurvy [1,3]. Without the antioxidative effects of ascorbic acid, biomolecules are subject to increased damage from reactive oxygen species which may lead to elevated inflammatory markers [3,5]. Irritation from subperiosteal or muscle hemorrhage in scurvy may also contribute to inflammatory marker elevations [5]. The most recent epidemiological studies on vitamin C deficiency (<11.4 μmol/L) in the pediatric population report a prevalence of 1.3% for those aged 6-11 years and about 2.7% for adolescents aged 12-19 [5]. However, these studies do not describe the epidemiology of symptomatic vitamin C deficiency. Therefore, we can not comment on the prevalence of scurvy.

Though uncommon in resource-rich countries, cases of scurvy have been documented in the United States in special populations of pediatric patients including those with autism spectrum disorder [5-7], developmental delay [5,8], or eating disorders [9]. These populations may have challenges consuming adequate amounts of vitamin C in their diet due to extreme food selectivity or poor dietary intake leading to malnutrition with micronutrient deficiency. 

## Case presentation

A seven-year-old nonverbal female with autism spectrum disorder presented with a month of limping that progressed to refusal to ambulate. The parents noted that she “cried out” when bearing weight on the left leg and had been experiencing night-time awakenings from pain. Of note, her diet primarily consisted of goldfish and dry cereal, and she had been recently referred to a feeding clinic to address difficulties in gaining weight. She was sent to the emergency department after her primary care physician obtained lower extremity x-rays which were concerning for a possible lytic mass on her left femur.

**Figure 1:**
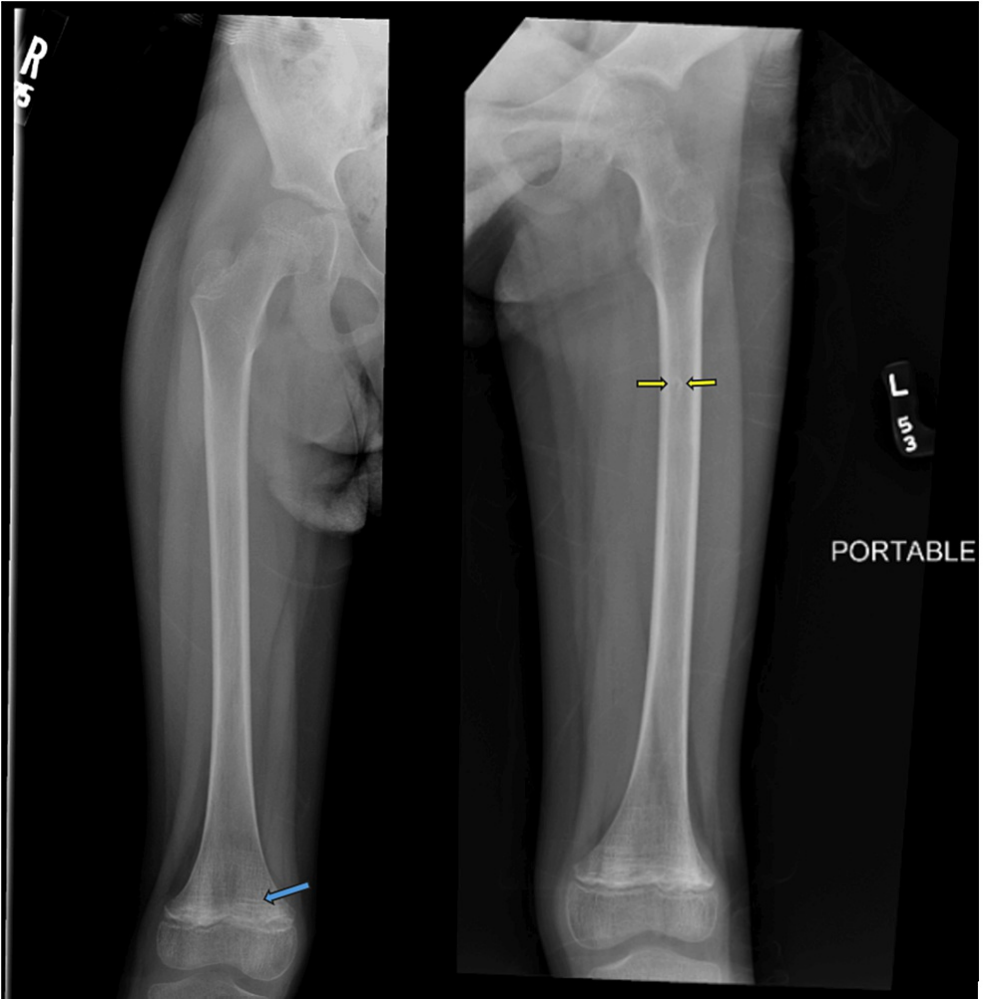
Plain radiograph (anteriorposterior views) of right and left femur. Bones are under mineralized subjectively and recovery lines (blue arrow) are present at distal femur bilaterally. There is a 5mm focal lucency (yellow arrows) in the left proximal femoral diaphysis with well-defined sclerotic margin, likely a benign non-ossifying fibroma. No suspicious osseous lesions are seen.

Examination revealed a thin young female who refused to stand. However, her lower extremities had a full range of motion, with no swelling or obvious deformities of hip or knee joints bilaterally. Initial labs were obtained and demonstrated elevated inflammatory markers, microcytic anemia, low alkaline phosphatase, and low vitamin D as shown in Table 1. Daily refeeding labs were initiated, and a plasma vitamin C level was sent. Repeat lower extremity radiographs indicated possible benign non-ossifying fibroma, osteopenia, and growth recovery lines, but were not concerning for lytic malignancy or fracture (Figure 1).

**Table 1:** Initial laboratory findings g/dL: grams per deciliter; fL: femtoliter; K/μL: 1000 per microliter; ng/mL: nanograms per milliliter; μg/dL: micrograms per deciliter; μmol/L: micromoles per liter; mm/hr: millimeters per hour; mg/dL: milligrams per deciliter; units/L: units per liter; units/mL: units per milliliter; μg/g: micrograms per gram; MCV: mean corpuscular volume; WBC: white blood cell; TIBC: total iron binding capacity; ESR: erythrocyte sedimentation rate; CRP: C-reactive protein; AST: aspartate aminotransferase; ALT: alanine aminotransferase; LDH: lactate dehydrogenase; IgA: immunoglobulin A; RF: rheumatoid factor; anti-CCP: anti-cyclic citrullinated peptide

Laboratory Study	Value	Reference Range
Hemoglobin	10.8 g/dL	11.5 - 15.5 g/dL
MCV	77.8 fL	80 - 100 fL
WBC count	8.78 K/μL	4.5 - 14.5 K/μL
Platelets	324 K/μL	150 - 400 K/μL
Ferritin	1426 ng/mL	13 - 150 ng/mL
TIBC	248 μg/dL	250 - 400 μg/dL
Iron	21 μg/dL	37 - 145 μg/dL
Vitamin D	24 ng/mL	30 - 100 ng/mL
ESR	33 mm/hr	0 - 10 mm/hr
CRP	0.53 mg/dL	<0.50 mg/dL
Alkaline phosphatase	107 units/L	142 - 335 units/L
Total bilirubin	0.2 mg/dL	0.0 - 1.2 mg/dL
LDH	213 units/L	135 - 250 units/L
Uric acid	4.4 mg/dL	2.7 - 6.4 mg/dL
Tissue transglutaminase IgA	<0.80 units/mL	0.0 - 4.99 units/mL
Fecal calprotectin	46 μg/g	<50 μg/g
Pancreatic elastase	>500 μg/g	>200 μg/g
RF	<10 units/mL	<14 units/mL
anti-CCP	<12 units/mL	<17 units/mL

Given her extremely selective diet, severe malnutrition, and ambulatory dysfunction, empiric treatment with oral vitamin C was initiated, and nutrition was consulted. After starting vitamin C treatment, the patient began to stand and walk short distances. Ambulatory function continued to improve over subsequent days. The patient was diagnosed with scurvy based on dramatic clinical improvement after starting treatment with vitamin C. At the time of discharge, vitamin C levels were undetectable at <5 μmol/L (levels <11.4 μmol/L considered deficient). Of note, repeat ferritin and c-reactive protein (CRP) levels were obtained on the day of discharge and had decreased to 980.3 ng/mL (reference range, 13-150 μmol/L) and <0.30 mg/dL (reference range, <0.50 mg/dL), respectively. On the day of discharge, the patient was ambulating effectively with a walker and she was referred to orthopedics and a local feeding clinic.

## Discussion

When a child presents with ambulatory dysfunction and elevated inflammatory markers, the differential diagnosis includes infectious (osteomyelitis, septic arthritis, transient synovitis), traumatic, autoimmune (juvenile idiopathic arthritis, chronic nonbacterial osteomyelitis), malignancy (osteosarcoma, Ewing sarcoma, leukemia), and nutritional (scurvy, rickets) etiologies. We therefore review the differential diagnosis for this patient with particular attention to our clinical reasoning.

Osteomyelitis can be acute or chronic and typically presents with erythema, warmth, and swelling at the site of infection along with systemic symptoms such as fever, chills, and malaise [10]. Elevated inflammatory markers or leukocytosis may also be present. Plain radiographs may show soft tissue swelling, osteopenia, osteolysis, and bony destruction. MRI has improved sensitivity and specificity for osteomyelitis compared to X-rays [10]. Our patient did not have systemic symptoms, localized findings on exam, or leukocytosis. While the radiographs showed evidence of possible osteopenia, there were no other findings on imaging suggestive of osteomyelitis. Transient synovitis is a common diagnosis given to pediatric patients with limp and can present with mild elevations of inflammatory markers [11]. Typically, the radiographs are normal with an ultrasound of the hip demonstrating a small effusion. In our case, the duration of the patient’s symptoms differed from the expected 7-10-day course in transient synovitis. Septic arthritis was also considered, but this infectious cause of limp often presents with fever, leukocytosis, and physical exam findings such as joint swelling, erythema, and limited range of motion, all of which were absent in our patient [11].

Malignancy was considered, but lower extremity radiographs were not suggestive of a malignant process and her complete blood count, lactate dehydrogenase, and uric acid levels did not raise concern for leukemia. The patient had no history of an injury preceding her limp and plain radiographs did not indicate fracture, which provided evidence against trauma as the etiology.

Juvenile idiopathic arthritis (JIA) is a diagnosis of exclusion with several subtypes [12]. Our patient lacked evidence of arthritis as she was without joint swelling, warmth, or erythema on physical exam. Rheumatoid factor and anti-cyclic citrullinated peptide (anti-CCP) serologies were obtained and were negative. These studies are only positive in a portion of polyarticular JIA cases and have more to do with prognosis than diagnosis when positive [12]. Although the ferritin elevations seen in our patient can also be present in systemic JIA (sJIA), she lacked evidence of widespread arthritis and extra-articular systemic manifestations seen in this condition such as intermittent fevers, lymphadenopathy, and migratory salmon pink rash [12]. Chronic, non-bacterial osteomyelitis (CNO) may also cause limp, elevated inflammatory markers, and a possible lytic lesion on radiographs in children [13]. However, CNO remains a diagnosis of exclusion, and clinical findings were sufficient to diagnose scurvy [13].

Our patient’s history of autism spectrum disorder and highly selective diet with poor growth raised our index of suspicion for vitamin deficiency as a cause for her ambulatory dysfunction. Her diet primarily consisted of goldfish and dry cereal and did not include common food sources rich in vitamin C such as oranges, tangerines, lemons, kiwis, melons, spinach, lettuce, broccoli, tomatoes, or potatoes [3]. Treatment dosing of vitamin C was initiated and led to a dramatic clinical improvement.

Scurvy can be diagnosed clinically with rapid clinical improvement after starting vitamin C treatment [6]. Plasma testing of vitamin C can confirm deficiency, but this test can be confounded by recent vitamin C intake [7]. Leukocyte vitamin C testing would be the most sensitive for scurvy and vitamin C deficiency, but this was not available at our hospital [1].

## Conclusions

This case highlights the importance of obtaining a dietary history in a pediatric patient with ambulatory dysfunction and a high index of suspicion for scurvy in special populations who may be at increased risk for vitamin deficiency due to a highly selective diet. Additionally, our case demonstrates that scurvy can present in pediatric patients as a limp or refusal to ambulate while lacking many of the classic clinical features associated with this syndrome. Elevated inflammatory markers should prompt consideration of a variety of etiologies causing ambulatory dysfunction, but it should not exclude scurvy as a possible diagnosis. 
